# New tools for automated cryo-EM single-particle analysis in RELION-4.0

**DOI:** 10.1042/BCJ20210708

**Published:** 2021-12-16

**Authors:** Dari Kimanius, Liyi Dong, Grigory Sharov, Takanori Nakane, Sjors H. W. Scheres

**Affiliations:** MRC Laboratory of Molecular Biology, Francis Crick Avenue, CB2 0QH Cambridge, UK

**Keywords:** computational biochemistry, cryo-electron microscopy, imaging techniques, structural biology

## Abstract

We describe new tools for the processing of electron cryo-microscopy (cryo-EM) images in the fourth major release of the RELION software. In particular, we introduce VDAM, a variable-metric gradient descent algorithm with adaptive moments estimation, for image refinement; a convolutional neural network for unsupervised selection of 2D classes; and a flexible framework for the design and execution of multiple jobs in pre-defined workflows. In addition, we present a stand-alone utility called MDCatch that links the execution of jobs within this framework with metadata gathering during microscope data acquisition. The new tools are aimed at providing fast and robust procedures for unsupervised cryo-EM structure determination, with potential applications for on-the-fly processing and the development of flexible, high-throughput structure determination pipelines. We illustrate their potential on 12 publicly available cryo-EM data sets.

## Introduction

Structure determination of biological macromolecules through single-particle analysis of cryo-EM images has recently reached a milestone by obtaining atomic resolution reconstructions [1,2]. With increasing resolutions, the applicability of cryo-EM structure determination continues to improve, and with many inexperienced scientists entering the field, the need for robust, easy to use image processing procedures is increasing. Moreover, atomic resolution structure determination opens up new avenues for cryo-EM structure-based drug design, which often requires high-throughput and automation to enable the screening of many candidate molecules.

The development of user-friendly cryo-EM image processing software has come a long way. Early software packages capable of performing cryo-EM structure determination by single-particle analysis, including SPIDER [3], IMAGIC [4] and the suite of MRC image processing programes [5], were mostly command-line driven and typically relied on extensive user experience to obtain good results. The development of graphical user interfaces (GUIs) and more integrated workflows in the EMAN software [6] reduced this requirement, making cryo-EM image processing accessible to more scientists. Developments in SPARX [7], BSOFT [8], FREALIGN [9] and XMIPP [10] also contributed to improved accessibility. The cryo-EM resolution revolution [11] further accelerated the focus on user-oriented software developments, with new software packages like SPHIRE [12], cisTEM [13] and the commercial cryoSPARC [14] implementing robust and easy-to-use pipelines for cryo-EM structure determination. In addition, overarching software developments like Appion [15] and Scipion [16] facilitated the combination of the different available software packages.

The first release of the RELION software coincided with the appearance of the first prototypes of direct electron detectors that would spark the resolution revolution [17]. RELION introduced a novel empirical Bayesian approach to single-particle analysis, with an explicitly regularized likelihood optimization target [18]. In the Bayesian framework, parameters for optimal filtering of the reconstruction are inferred from the data, thus removing the need for user expertise to tune related parameters in alternative softwares. Not only did the Bayesian approach lead to higher quality reconstructions; it also represented a step-change in software accessibility that expedited a rapid expansion of the field once direct detectors became commonly available [19].

More recently, automation of large parts of the cryo-EM structure determination pipeline has received increased attention. In particular, various unsupervised protocols for the earlier stages of image processing, including motion correction in movies, contrast transfer function (CTF) parameter estimation and particle picking, have been introduced, for example in FOCUS [20], SCIPION [21], WARP [22], tranSPHIRE [23], SPREAD [24] and cryoFLARE [25]. Automated on-the-fly processing of cryo-EM data allows spotting problems in the data while they are being acquired, thus providing opportunities to change data collection and save valuable time on the microscope. In addition, their standardized procedures lower the barriers for novel users and facilitate the development of high-throughput structure determination pipelines.

This paper describes new tools for single-particle analysis in RELION-4.0 that aim to make unsupervised cryo-EM structure determination faster, more robust and easier to automate.

## Methods

### VDAM: variable-metric gradient descent with adaptive moments estimation

#### Regularized likelihood optimization

We briefly recapitulate the regularized likelihood optimization algorithm that underlies classification and refinement procedures in RELION [17,18].

Let $X=x_{1},\ldots ,x_{N}\in C^{L}$ denote the Fourier transforms of the experimental particle images. Each particle image is a noisy 2D projection of a rotated and translated volume, out of an ensemble of unknown volumes with 3D Fourier transforms $V=v_{1},\ldots ,v_{K}\in C^{M}$, typically referred to as classes. We assume

1 $$x=H^{q}v+e,$$

where $H^{q}\in C^{L\times M}$ is a complex valued matrix that takes a 2D slice out of the 3D Fourier transform after applying the relevant composite transformation *q* ∈ *Q* : = *SE*(3), consisting of a rotation and translation, as well as a (given) CTF. We assume uncorrelated Gaussian noise, or $e_{i}\in C^{L}∼CN(0,\sigma )$, as well as uncorrelated Gaussian signal, or $v\in C^{M}∼CN(0,\tau )$, where both $\sigma \in R_{+}^{L}$ and $\tau \in R_{+}^{M}$ are diagonal co-variance matrices.

We then seek the *maximum a posteriori* (MAP) estimate of V by maximizing the following regularized likelihood function:

2 $$L(V,X)\;:\;=\sum_{i=1}^{N}\log\sum_{k=1}^{K}\int_{Q}P(x_{i}|v_{k},q)P(q)dq+\sum_{k=1}^{K}\log P(v_{k}),$$

where we have assumed that X contains independent observations, and we have marginalized over the nuisance variables, through an integration over *Q* and a summation over *k*. The likelihood term is calculated as $P(x_{i}|v,q)=CN(|x_{i}-H_{i}^{q}v|,\sigma )$; and the prior term as P⁡(v)=CN(0,τ). P⁡(q) expresses information about the prior probability of the transformations, e.g. a 2D Gaussian distribution for the translations and typically a uniform distribution for rotations.

To find the MAP estimate of V, we use the Expectation–Maximization algorithm, where we denote each iteration with the index (*n*). Starting from an initial guess, $V^{\left(0\right)}$, we apply a fixed-point iteration approach by fixing V and solving $\nabla_{v}L(V,X)=0$ for the parameters of a particular *v*. First, in the Expectation step we calculate the gradient of (2) with respect to *v*_*k*_:

3 $$\nabla_{v_{k}}L(V,X)=\sum_{i=1}^{N}\int_{Q}P_{k}(q|V,x_{i})[{H_{i}^{q}}^{*}\sigma^{-2}(x_{i}-H_{i}^{q}v_{k})]dq-\tau^{-2}v_{k},$$

with

4 $$P_{k}(q|V,x_{i})=\frac{P(x_{i}|v_{k},q)P(q)}{\sum_{k^{\prime }=1}^{K}\int_{Q}P(x_{i}|v_{k^{\prime }},q)P(q)dq}.$$

Then, in the Maximization step we solve for the parameters of a particular *v*_*k*_, which yields the closed-form solution:

5 $$v_{k}^{\left(n+1\right)}\leftarrow \frac{B_{k}^{\left(n\right)}}{F_{k}^{\left(n\right)}+\tau^{-2}}$$

where the division is to be evaluated element-wise, and where

6 $$\begin{array}{cc} B_{k}^{\left(n\right)} & =\sum_{i=1}^{N}\int_{Q}P_{k}(q|V,x_{i})[{H_{i}^{q}}^{*}\sigma^{-2}x_{i}]\in C^{M},\\ F_{k}^{\left(n\right)} & =\sum_{i=1}^{N}\int_{Q}P_{k}(q|V,x_{i})[\mathrm{diag}({H_{i}^{q}}^{*}\sigma^{-2}H_{i}^{q})]\in R_{+}^{M}. \end{array}$$

#### Variable-metric gradient descent

Optimization by gradient descent is an alternative to the Expectation–Maximization algorithm, where the update formula is generally a step in the direction of the negative gradient weighted with the learning rate, η∈R:

7 $$v_{k}^{\left(n+1\right)}\leftarrow v_{k}^{\left(n\right)}-\eta (G_{k}^{\left(n\right)}+\tau^{-2}v_{k}^{\left(n\right)})$$

with $G_{k}^{\left(n\right)}\;:\;=-\nabla_{v_{k}}L(V^{\left(n\right)},X){|}_{v_{k}=v_{k}^{\left(n\right)}}$, as in (3). In this approach, the summation over *i* in (3) and (6) are typically carried out over a random subset of the data set, called a mini-batch, which is changed at each iteration.

We notice that the Expectation–Maximization algorithm can be viewed as a variable-metric gradient descent (VMGD) algorithm, where the gradient in the update formula has been modified with a positive definite projection matrix, *D*^(*n*)^, which changes every iteration [26]. Applying this to the GD update formula in (7) gives

8 $$v_{k}^{\left(n+1\right)}\leftarrow v_{k}^{\left(n\right)}+\eta D_{k}^{\left(n\right)}[G_{k}^{\left(n\right)}-\tau^{-2}v_{k}^{\left(n\right)}]$$

We seek a positive definite matrix *D*^(*n*)^ that equates the gradient descent update to the Expectation–Maximization update. From (5) and (8) we get

9 $$\frac{B_{k}^{\left(n\right)}}{F_{k}^{\left(n\right)}+\tau^{-2}}=v_{k}-\eta D_{k}^{\left(n\right)}[G_{k}^{\left(n\right)}+\tau^{-2}v_{k}].$$

Solving for *D*, yields

10 $$D_{k}^{\left(n\right)}=\mathrm{diag}\{[F_{k}^{\left(n\right)}+\tau^{-2}{]}^{-1}\}.$$

Inserting this into (8) yields the following update formula for the VMGD algorithm:

11 $$v_{k}^{\left(n+1\right)}\leftarrow v_{k}^{\left(n\right)}-\eta \frac{G_{k}^{\left(n\right)}+\tau^{-2}v_{k}^{\left(n\right)}}{F_{k}^{\left(n\right)}+\tau^{-2}}$$

Note that, if the gradient was calculated over the entire data set, *η* can now be set to 1.0, since the gradient is rescaled by *D* to fit the Expectation–Maximization step size. However, if updates are performed with mini-batches, *η* should still be smaller than 1.0, because the estimated *G* from a subset is noisier. In our implementation, the default values for *η* range 0.1–0.9, depending on the stage of reconstruction.

If a Fourier shell correlation (FSC) can be calculated that assesses the signal-to-noise for $v_{k}^{\left(n\right)}$, then, using equations (9) and (10) in [17], (11) can be rewritten as

12 $$v_{k}^{\left(n+1\right)}\leftarrow v_{k}^{\left(n\right)}-\eta \left[{\mathrm{FSC}}_{k}^{\left(n\right)}\frac{G_{k}^{\left(n\right)}}{F_{k}^{\left(n\right)}+\epsilon_{1}}+(1-{\mathrm{FSC}}_{k}^{\left(n\right)})v_{k}^{\left(n\right)}\right]$$

where *ε*_1_ is a constant added to improve numerical stability.

In our implementation, we calculate ${\mathrm{FSC}}_{k}^{\left(n\right)}$ using two separately reconstructed versions of *v*_*k*_, each from one half of the data set X. Additionally, we define the rescaled gradient $\hat{G}\;:\;=G/(F+\epsilon_{1})$, which is invariant to the mini-batch size, for small values of *ε*_1_.

#### Adaptive learning rate

We also implemented an adaptive learning rate method, similar to methods commonly used in deep-learning, including Adam [27], Adagrad [28] and ADADELTA [29]. Typically, these methods accumulate two gradient moments, through running averages: $m\in C^{M}$ tracks the momentum of the gradient (first moment), while $u\in R^{M}$ tracks an estimate of the noise or error amplitude in the gradient (second moment). In particular, the Adam optimizer, tracks |*G*|^2^ as the second moment. Instead, we calculate two gradients, $G_{h_{1}}$ and $G_{h_{2}}$, for two separate halves of the data set, h_1_ and h_2_, and accumulate these into two separate first moments. The second moment, which is shared among the two halves, tracks $|G_{h_{1}}-G_{h_{2}}{|}^{2}$. Additionally, we avoid having to track *F* separately by tracking the rescaled gradient $\hat{G}$ instead. Thereby, we consider the following three running averages for each *k*:

13 $$\begin{array}{c} m_{h_{1}}^{\left(n+1\right)}\leftarrow \beta_{1}m_{h_{1}}^{\left(n\right)}+(1-\beta_{1}){\hat{G}}_{h_{1}}^{\left(n\right)}\\ m_{h_{2}}^{\left(n+1\right)}\leftarrow \beta_{1}m_{h_{2}}^{\left(n\right)}+(1-\beta_{1}){\hat{G}}_{h_{2}}^{\left(n\right)}\\ u^{\left(n+1\right)}\leftarrow \beta_{2}u^{\left(n\right)}+(1-\beta_{2})|{\hat{G}}_{h_{1}}^{\left(n\right)}-{\hat{G}}_{h_{2}}^{\left(n\right)}{|}^{2}. \end{array}$$

The update formula for each half data set then becomes:

14 $$v_{k,h}^{\left(n+1\right)}\leftarrow v_{k,h}^{\left(n\right)}-\eta \left[{\mathrm{FSC}}_{k}^{\left(n\right)}\frac{m_{k,h}^{\left(n\right)}}{\sqrt{u_{k}^{\left(n\right)}}+\epsilon_{2}}+(1-{\mathrm{FSC}}_{k}^{\left(n\right)})v_{k,h}^{\left(n\right)}\right]\;\mathrm{for}\;h=h_{1},h_{2},$$

where *ε*_2_ is a suitably small constant. In our implementation, *β*_1_ = 0.9 and *β*_2_ = 0.999.

Excluding the regularization term in (12) from the running averages in (13) serves to decouple the effects of regularization and the learning rate, which has been shown to improve convergence efficiency for the Adam optimizer [30].

#### Replacing inactive classes

In previous releases of RELION, especially in 2D classification, many classes would converge to contain no or very few particles. This represented a waste of computational resources and often resulted in suboptimal classification of structural variability in the data. To address this issue we here propose an algorithm that, throughout the gradient optimization, substitutes classes with small likelihood probabilities with classes that exhibit large variability. This approach is inspired by methods used in the class of artificial neural network algorithms known as self-organizing maps and neural gases [31]. At the end of each mini-batch, before the gradient update is applied, we select class *a*, which is the class with the smallest likelihood probability, $P(X|v_{a})$. Next, we select class *b*, which is the class with the largest $|m_{b}/(\sqrt{u_{b}}+\epsilon ){|}^{2}$. We then update *v*_*a*_ and its corresponding moments as we would have done for *v*_*b*_ using (13) and (14), and we keep *v*_*b*_ the same as it was in the previous mini-batch. This effectively substitutes the smallest class with a numerically different version of the class that is changing the most. The class substitutions are only performed if $P(X|v_{a})<\rho /K$, where *ρ* is a user-defined constant, called the *class inactivity threshold* (see section ‘2D classification with the VDAM algorithm’).

#### Implementation details

In previous releases of RELION, the initial references for 2D classification and 3D initial model generation were initialized from averages of random subsets of the particles in random orientations. In RELION-4.0, the default is to start from randomly positioned Gaussian blobs that are different for each class. The primary purpose of this initialization is to diversify the classes early in the classification, thus leading to faster convergence and the recovery of more class variability.

To further reduce computational costs for both 2D classification and 3D initial model generation, VDAM optimizations are started with a high learning rate of 0.9. By default, the learning rate is then gradually reduced to 0.3 for 2D classification and to 0.5 for 3D initial model generation. In addition, calculations are started from relatively small mini-batches: 0.5% of the total data set size (with a minimum of 200 particles, and a maximum of 10 000 particles for 2D classification and a maximum of 5000 for 3D initial model generation). After the initial stage the learning rate is reduced to 0.3 for 2D classification and to 0.5 for 3D initial model generation, and the mini-batch size is increased to 5% and 10% of the data set size, respectively (with a minimum of 1000 particles, and a maximum of 100 000 particles for 2D classification and 50 000 particles for 3D initial model generation).

Although most of the data sets tested in this paper converge after 100 mini-batches, in order to obtain good results for a larger number of data sets, we set the default on the GUI to 200 mini-batches, and ran all calculations in this paper using 200 mini-batches. In this way, the total number of passes through the entire data set, i.e. epochs, is five or less, resulting in a major speed-up compared with the 25 full iterations that are done by default using the EM algorithm. In the final iteration, a final pass through the entire data set is performed, where only P⁡(X∣k) for each class *k* is calculated, further saving time compared with a normal epoch. In addition, we noticed that the VDAM algorithm is less sensitive to truncation of the marginalization (i.e. skipping those orientations from the integral in equation (2) with low probabilities) than the EM algorithm, leading to additional increases in speed, in particular during the early stages of refinement.

### Class ranker: automated 2D class selection

**Figure 1.**
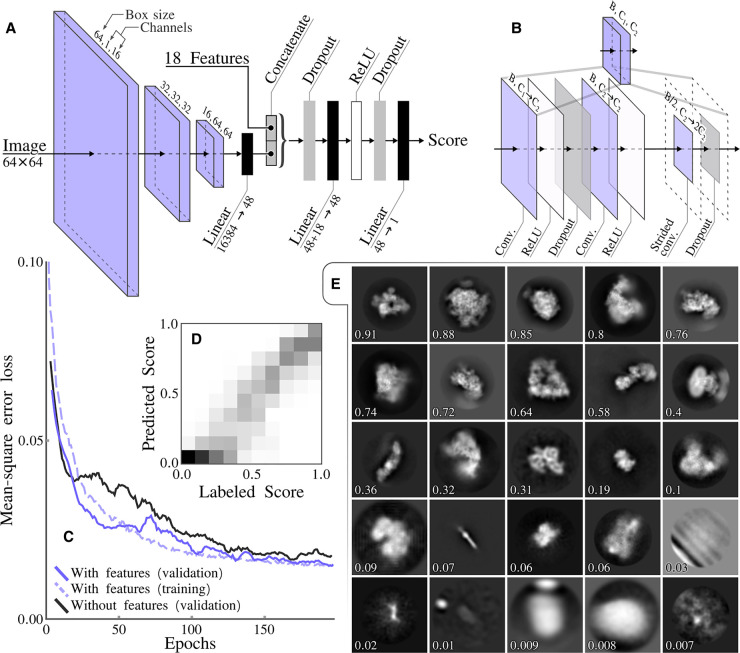
Class ranker neural network architecture and results. (**A**) The overall architecture of the scoring network, which consists of three CNN blocks and a final feed forward network that also incorporates the 18 features. (**B**) The CNN block architecture that incorporates three convolutional layers. The initial convolutional layer, maps the input channels count *C*_1_ to the intermediate channels count *C*_2_ and the final layer preforms a down sampling of the box size through a strided convolution and doubles the number of channels. (**C**) The mean-square error loss during training, comparing with and without features. (**D**) A confusion matrix showing labeled scores versus predicted scores, with bins of 0.1. (**E**) Example of classes with their predicted score.

The selection of particles that give rise to 2D class average images with recognizable protein features is often used to discard suboptimal particles from cryo-EM data sets. The selection of suitable 2D classes was done interactively in previous releases of RELION. RELION-4.0 contains a new programe called relion_class_ranker that automates 2D class selection. This programe predicts a score for each class by combining the output of a convolutional neural network that acts on the 2D class average images with 18 features (Figure 1A,B).

**Table 1.** Test data set characteristics

Data set	EMPIAR entry	Nr micrographs	Super-resol?	Voltage (V)	$C_{s}$	Phase plate?	Pixel size (Å)	Symmetry	Particle diameter (Å)
ribosome	10 028	1082	No	300	2.7	No	1.34	C1	320
TRPV1	10 059	1200	No	300	2.7	No	1.22	C4	150
TcdA1	10 089	97	No	300	2.7	No	1.14	C5	280
apoF	10 146	20	No	300	0.01	No	1.5	O	130
ribo-VPP	10 153	315	Yes	300	0.01	Yes	0.545	C1	320
aldolase	10 181	659	Yes	200	2.7	No	0.46	D2	180
γ-sec	10 194	2922	Yes	300	2.7	No	0.7	C1	150
β-gal	10 204	24	No	200	1.4	No	0.885	D2	180
CMV	10 205	5619	No	300	2.7	No	1.065	I1	330
GDH	10 217	2491	No	300	2.7	No	0.66	D3	150
CB1	10 288	2754	No	300	2.7	No	0.86	C1	160
INX6	10 290	497	No	300	2.7	No	1.23	C8	150

ribosome: *Plasmodium falciparum* 80S ribosome [39]; TRPV1: transient receptor potential channel V1 [40]; TcdA1: Tripartite Tc toxin subunit A [12]; apoF: apoferritin; ribo-VPP: ribosome collected on a Volta phase plate [41]; aldolase: Rabbit muscle aldolase [42]; γ-sec: γ-secretase [43]; β-gal: β-galactosidase; CMV: cowpea mosaic virus [44]; GDH: glutamate dehydrogenase; CB1: cannabinoid receptor 1G [45]; INX6: innexin-6 hemichannel [46].

**Table 2.** Comparison between the two algorithms for 2D classification

	Selected particles		Time (hh:mm)		Resolution (Å)	
	EM	VDAM	EM	VDAM	EM	VDAM
TRPV1	250 182	203 740	03:26	00:50	3.2	3.3
γ-sec	340 535	350 583	13:49	02:56	3.8	3.8
GDH	476 531	476 559	05:01	00:51	2.5	2.4
CB1	587 385	500 473	12:18	02:46	3.3	3.3
INX6	88 855	89 141	02:04	00:28	4.0	4.0

Good classes were selected manually, and the selected subset was further processed in auto-refine. The number of manually selected particles are shown for each data set and algorithm, as well as the execution time for 2D classification and the final resolution achieved with auto-refine using the subset from the respective subsets.

The convolutional neural network takes as input individual 2D class average images, cropped to contain only the area defined by the circular mask used in the 2D classification, and rescaled to 64 × 64 pixels. The feature vector is calculated for each class from RELION’s metadata of the 2D classification job, including the estimated accuracies of rotational and translational alignments, the estimated resolution (1/*d* in 1/Å) and a so-called weighted resolution, which is calculated as *d*^2^/ln*N*, where *N* is the number of particles assigned to the class. It also contains features that are calculated from the 2D class average images, in particular the first to fourth moments of density values inside an automatically determined mask for the protein region, the solvent region, and for a ring around the outer diameter of the mask that has been applied to the 2D class average images. The combined output from the convolutional neural network and the feature vector is passed through two fully connected layers, with non-linear (ReLU) activation functions between the layers, to predict a single, floating point value, score for each 2D class.

The network in the relion_class_ranker program was trained on 18 051 2D class average images from 233 RELION 2D classification jobs that were performed at the MRC-LMB over a period of approximately 4 years. Each of the jobs was assigned a job score, ranging from zero to one, and within jobs the class averages images were manually divided into four categories depending on their quality. For each 2D class, the combination of its job score, its category assigned and its estimated resolution compared with the best resolution in its 2D classification job, were used to calculate a target class score, ranging from zero to one. The target scores were intended to represent a ranking over all classes in the training set, with a score of one representing the best classes from the best 2D classification jobs, and a score of zero representing the worst classes. The network was implemented and optimized with the Adam optimizer [27] for 200 epochs in pytorch [32], using a mean-squared error between predicted and assigned scores. All 18 051 class average images, plus their metadata from the 2D classification jobs and their assigned class scores are publicly available through the EMPIAR data base (entry-ID 10812). The code used to optimize and execute the neural network are available from the RELION github pages.

### Schemes: planning and execution of multiple jobs

RELION’s pipeliner organizes the execution of RELION jobs, which represent individual tasks, and often the execution of an individual command-line program, in the overall structure determination workflow. The pipeliner also keeps track which jobs’ output files are used as input for other jobs, thus building a directional graph of the processing workflow [33].

RELION-4.0 implements new functionality to plan the execution of multiple jobs in advance, including functionality to execute branched decision trees, where decisions to follow one branch of sequential jobs or another are made on-the-fly. A series of planned jobs, possibly including multiple branches, is called a *Scheme*.

To allow for flexibility in the design of the execution of multiple jobs, Schemes implement different types of variables: *stringVariables*, *booleanVariables* and *floatVariables*. The values of these variables can be changed through the execution of so-called *Operators* that form part of the Scheme framework. Multiple Operators have been implemented, for example to perform simple mathematical operations on floatVariables; to perform logical operations on booleanVariables; and to perform text modifications on StringVariables. In addition, Operators exist for reading metadata values from RELION star files; for file handling operations; for sending emails and for waiting a pre-determined amount of time. A full description of available Operators is available from the RELION documentation, which has been rewritten, and is available from: https://relion.readthedocs.io/en/release-4.0/.

Schemes can be thought of in terms of a directional graph, where the nodes of the graph are either jobs or Operators. *Edges* connect two subsequent nodes, while *Forks* connect one input node to two possible output nodes. Each Fork has an associated booleanVariable, whose value determines which of the two output nodes is chosen upon execution of the Scheme. The topology of the graph inside a Scheme can be cyclical, thereby enabling repetitive execution of jobs inside loops.

Schemes are defined by a scheme.star file that describes the different jobs, Operators, Variables, Edges and Forks. The scheme.star file is stored inside a Schemes/schemename directory, which itself is inside the standard RELION Project directory. The Scheme directory also contains subdirectories for each of the RELION jobs that form part of the Scheme. These subdirectories each contain a file called job.star that contains the parameter values for that job. The Scheme framework is flexible, in that users can define their own Schemes by manually editing the corresponding star files. The job.star files for individual jobs can also be saved through the Jobs menu on the RELION-4.0 main GUI.

Schemes are executed through the relion_schemer program, which launches the jobs, and keeps track of the current status of the Scheme and the values of all its variables. It can also be used to abort a running Scheme, to change its current variables or the parameters of its RELION jobs and to re-start from the point where it was previously aborted. If any RELION job parameters were changed, the relion_schemer program will re-execute those jobs from scratch, whereas jobs that are unaffected by the changes will continue from where they were halted.

**Figure 2.**
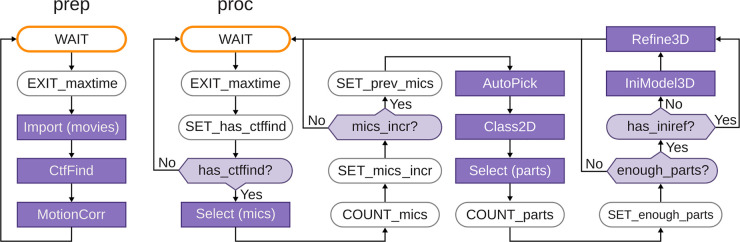
**Schematics of the prep and proc Schemes that form part of the relion_it.py approach for automated, on-the-fly processing.** Scheme operators are shown with rounded boxes, RELION jobs with purple boxes; edges with arrows and forks with light purple diamond shapes. For forks, the BooleanVariable that controls its outcome is indicated in the center of the diamond. The WAIT operator waits for a defined time since it was last executed; the EXIT_maxtime operator terminates the Scheme after a defined time since the Scheme was started; the SET_has_ctffind operator sets BooleanVariable has_ctffind to true if the STAR file generated by the CtfFind job of the prep Scheme exists; the COUNT_mics operator sets the current number of micrographs to the number selected in the job above it; the SET_mics_incr sets BooleanVariable mics_incr to true if the current number of selected micrographs is larger than the previous number of micrographs (which is initialized to zero); the SET_prev_mics operator sets the previous number of micrographs to the current number of selected micrographs; the COUNT_parts operator sets the current number of particles to the number of selected particles in the job above it. The SET_enough_parts operator sets BooleanVariable enough_parts to true if the current number of selected particles is larger than a user-specified minimum.

RELION-4.0 includes two example Schemes, called prep and proc. The prep Scheme imports micrograph movies and performs motion correction and CTF estimation. The proc Scheme selects micrographs based on their estimated CTF resolution limit, performs automated particle picking (using either RELION’s Laplacian-of-Gaussian (LoG) approach or Topaz [34]), 2D classification, automated 2D class selection, 3D initial model generation and 3D refinement. A flowchart of both Schemes, depicting all corresponding RELION jobs and Scheme Operators is shown in Figure 2.

**Figure 3.**
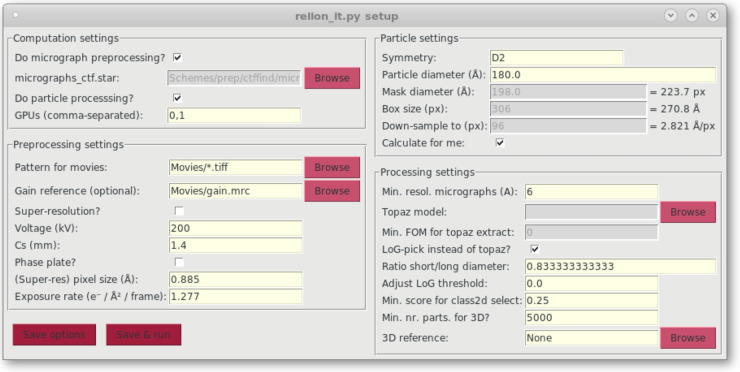
GUI of the relion_it.py script for automated execution of the prep and the proc Schemes.

The python script relion_it.py, which already existed in RELION-3, has been modified to work with Schemes in RELION-4.0. The modified script launches a GUI (see Figure 3) to gather parameter input from the user and then executes the prep and the proc Schemes to process cryo-EM data sets in an unsupervised manner. In addition, a new GUI called relion_schemegui.py has been written to facilitate the monitoring of Schemes during their execution, as well as their aborting, changing of variables and re-starting.

### MDCatch: integration with the microscope

**Figure 4.**
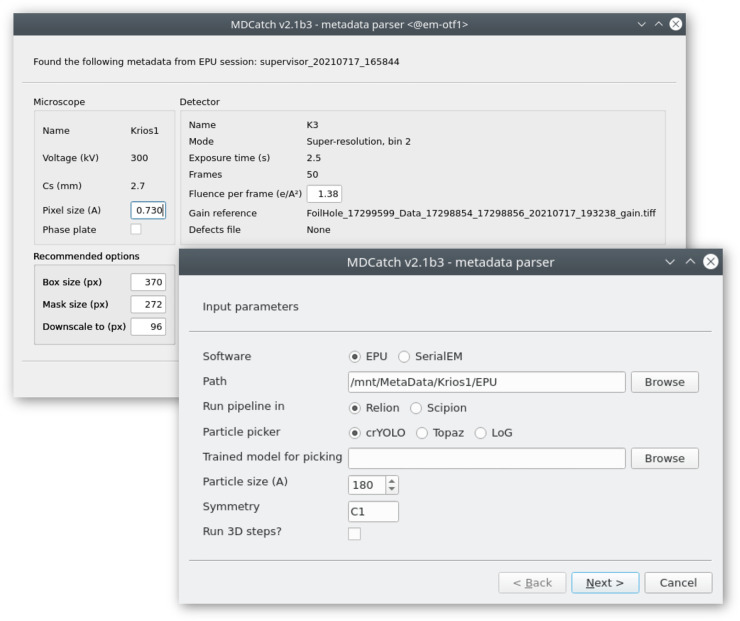
GUI of the MDCatch utility for automated fetching of microscope metadata and launching of on-the-fly image processing.

To simplify launching of on-the-fly image processing and minimize user input errors we implemented MDCatch, a graphical tool that extends relion_it.py functionality by linking microscope data acquisition with the execution of RELION-4.0 Schemes. MDCatch is written in Python3 and PyQt5 and provides a simple GUI (Figure 4) that fetches acquisition metadata from a running EPU (Thermo Fisher Scientific) or SerialEM [35] session, and launches a pre-defined image processing pipeline. Besides RELION-4.0 Schemes, MDCatch also works with Scipion 3 workflows [21]. MDCatch supports parsing of metadata from different file formats (EPU’s XML, SerialEM’s MDOC, MRC, TIF) and associates this information with other microscope parameters (detector type, MTF, gain reference etc.) that can be configured in advance. In cases where existing metadata is not sufficient, users can manually input missing information. MDCatch was designed to be installed on a computer that has access to both the raw data (movies) and its associated metadata, e.g. an EPU session folder. For SerialEM data acquisitions, both movies and MDOC metadata files are expected to be in the same directory.

Together with MDCatch we provide example pipeline templates for both RELION and Scipion, including the prep and proc Schemes described above. By default, users are presented with a choice between three particle pickers: the LoG approach in RELION, crYOLO [36] or Topaz [37]. Upon execution of MDCatch, the fetched metadata are saved in a text file and the image processing progress can be monitored with existing project utilities in RELION or Scipion.

MDCatch is distributed separately from RELION-4.0, under a free, GPLv3 software license, and its code and documentation are available at https://github.com/azazellochg/MDCatch.

## Results

### Optimization of the neural network in relion_class_ranker

During exploration of network architecture and optimization procedures, 5850 2D class average images, from 73 2D classification jobs, were set aside as a validation set to monitor overfitting. Because the final network architecture and optimization procedure did not induce noticeable amounts of overfitting (Figure 1C), a final optimization round was performed using all 18 051 classes. The resulting network had a mean-square error loss of 0.015 on the predicted class scores. Optimization of a network where all feature values were set to zero led to a mean-square error loss of 0.017, indicating that the features provided useful information in the scoring process. Analysis of 2D histograms of the assigned and predicted class scores (Figure 1D) and manual assessment of the predicted scores (Figure 1E) confirmed that the final network produces useful predictions over the full range of assigned class scores. The optimized network was further tested as part of the automated processing of 12 test data sets through the Schemes and relion_it.py approach, as described below.

### Automated processing with Schemes and relion_it.py

To test the procedures for automated single-particle analysis in RELION-4.0, we processed 12 data sets from the EMPIAR data base [38], using default parameters from relion_it.py, except for the experiment-specific parameters and the particle diameter as shown in Table 1. The 12 data sets were selected at the start of the project; no data sets were added or removed during the project. Motion correction for movies of these data sets were performed in RELION’s own implementation of the MotionCor2 algorithm [47]. CTF estimation was performed in CTFFIND4 [48]. Motion-corrected micrographs and extracted particles were saved in IEEE 754 16-bit float MRC format (mode 12), a new feature in RELION-4.0 to save a factor of two in required disk space. The proc Scheme was used for automatically processing the data, up to 3D initial model generation and refinement of downscaled particle images.

**Figure 5.**
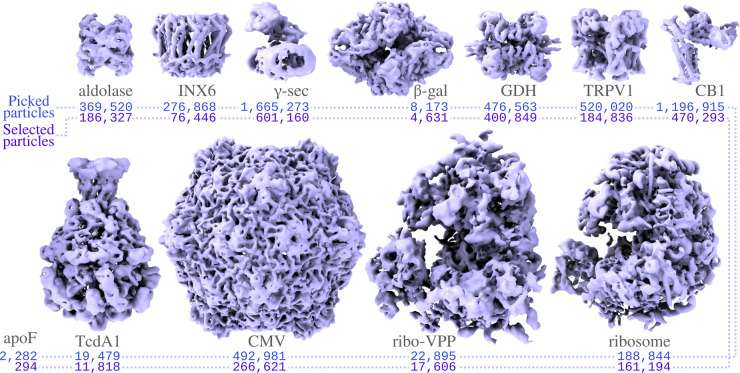
Automated structure determination for the test data sets. For each data (see Table 1) the reconstruction after refinement with the downsampled particles is shown, together with the number of auto-picked particles and the number of selected particles. Although not shown here, for the GDH, TRPV1 and aldolase data sets, initial model generation does some times get stuck in local minima (see section ‘Initial 3D model generation with the VDAM algorithm’ for more details). No map was reconstructed for apoF.

Only micrographs with resolutions beyond 6 Å, as estimated by CTFFIND-4, were included in the processing. For all data sets, except for the ribosome data set collected with a phase plate (ribo-VPP; EMPIAR-10153), particle picking using the pre-trained model in Topaz yielded reasonable results. For the ribo-VPP data set, the unusually strong contrast in the phase plate images yielded suboptimal results in Topaz, and we used the LoG-picker in RELION instead. All particles were extracted in the box sizes suggested by relion_it.py, i.e. 1.5 times the particle diameter, and downscaled to pixel sizes in the range of 2.8–3.5 Å (with the exact pixel size depending on favorable downscaled image sizes for the fast Fourier transform algorithm). The extracted particles were subjected to 2D classification with 100 classes, using the VDAM algorithm, followed by automated class selection in relion_class_ranker with a default minimum class score of 0.15. Finally, the selected particles were subjected to 3D initial model generation in symmetry group C1, again using the VDAM algorithm, with three classes, followed by 3D auto-refinement of the largest class after automated detection and alignment of the symmetry axes. Figure 5 gives an overview of the results.

For all data sets, except the apoferritin data set (EMPIAR-10146), 2D classification with the VDAM algorithm (see section ‘2D classification with the VDAM algorithm’) provided adequate information to assess the quality of the data and the class ranking successfully identified suitable 2D class averages (see section ‘Automated 2D class selection with relion_class_ranker’). Dense packing of the apoferritin particles in the micrographs caused the appearance of density for neighboring particles in the 2D class averages, which resulted in too low class scores. Because only 294 apoferritin particles were selected, no 3D model generation was attempted. For all other data sets, correct reconstructions could be obtained in a fully automated manner with resolutions close to the Nyquist limit for the downscaled particles (but also see section ‘Initial 3D model generation with the VDAM algorithm’).

### 2D classification with the VDAM algorithm

**Figure 6.**
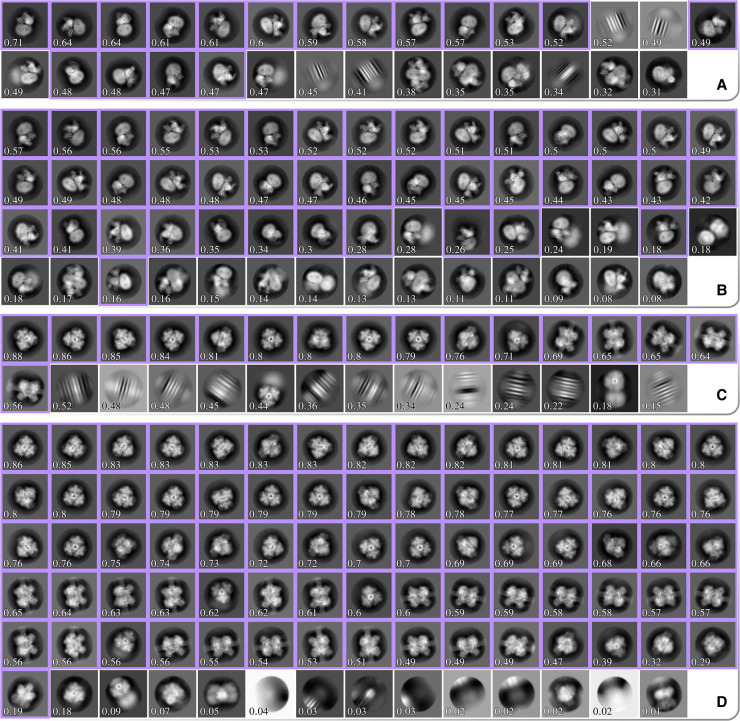
**All significant 2D class averages from four different classification runs.** (**A**) and (**B**) show results for the GDH data set classified using the EM and VDAM algorithm, respectively. (**C**) and (**D**) show results for the CB1 data set classified using the EM and VDAM algorithm, respectively. Classes are sorted according to their score from the relion_class_ranker program, which is also shown for each class. Classes that were manually selected for subsequent 3D auto-refinement are highlighted in purple.

Figure 6 shows two example comparisons between 2D classifications with the VDAM and the EM algorithms: for the GDH and CB1 data sets. For each run, we manually selected the best classes (highlighted in purple in Figure 6 for GDH and CB1), and subsequently used the corresponding sets of particles for 3D auto-refinement to asses the relative quality of the classified subsets. Computations were performed on an Intel Xeon Gold 6242 and four NVIDIA GeForce RTX 2080Ti GPUs. All VDAM calculations were performed with the default class inactivity threshold of 0.1.

Table 2 shows the results for five test data sets. Each run consists of 25 EM and 200 VDAM iterations, which corresponds to 25 and approximately 6 epochs, respectively. The final epoch for the VDAM algorithm is only performed to assess particle class assignment, and is thus faster. On average, the VDAM algorithm is a factor of 5 faster compared with the EM algorithm for 2D classification, without affecting the quality of the selected particles, as measured by the final resolution after auto-refinement.

### Automated 2D class selection with relion_class_ranker

**Figure 7.**
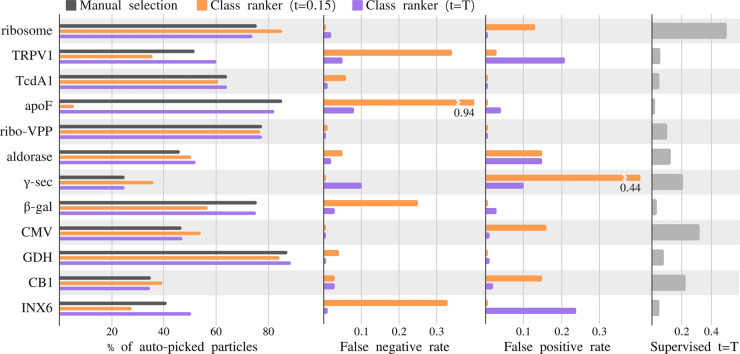
**Analysis of the automated 2D class selection.** For the 12 test data sets, the charts on the left show the percentage of particles after manual selection (gray), after automated selection with a default threshold of 0.15 (orange), and automated class selection with a supervised threshold (purple). The center two panels show the false positives rate, i.e. number of particles selected by the class ranking procedure, but not by manual selection, divided by the number of selected particles in the manual selection, and the false negative rate, i.e. number of particles selected by manual selection, but not by the class ranking procedure, divided by the number of selected particles by manual selection, for the default (purple) and the supervised (orange) thresholds. The panel on the right shows the value of the supervised threshold (*t* = *T*).

The predicted class score of the relion_class_ranker program was designed to be on an absolute scale, ranging from a value of zero for useless classes to a value of one for the best classes from the best data sets. Therefore, because some data sets are better than others, the threshold for class selection may need to be adjusted in line with the expected quality of the 2D class average images for a given data set. Figure 7 shows an evaluation of the quality of the particle selection for all 12 test data sets, by comparing the selections based on the indicated class score threshold (*t*) with a manual selection of suitable classes. Quality is measured in terms of the false positive rate (FPR) and the false negative rate (FNR) of the particles from the selected 2D classes, where the particles from the manually selected classes are considered the correct ones. To reflect that the threshold value may be changed based on the expected quality of each data set, besides reporting the results for the default score threshold of 0.15 used in relion_it.py, this table also shows the results for a freely chosen, i.e. supervised threshold value (*t* = *T*) for each data set.

For the ribosome, CMV, CB1 and *γ*-sec data sets, a higher threshold than the default leads to better results, although only for the *γ*-sec data set the FPR is higher than 0.2 using the default threshold. For the TRPV1, apoF, *β*-gal and INX6 data sets, a lower threshold yields better results, with the default threshold yielding FPRs of 0.25 or higher. Nevertheless, as pointed out above, even when using the default threshold of 0.15, the particle selection for all data sets, except apoF, allowed *de novo* reconstruction of a correct 3D map. Using the supervised threshold, for all data sets the FPR and the FNR are below 0.25.

### Initial 3D model generation with the VDAM algorithm

**Figure 8.**
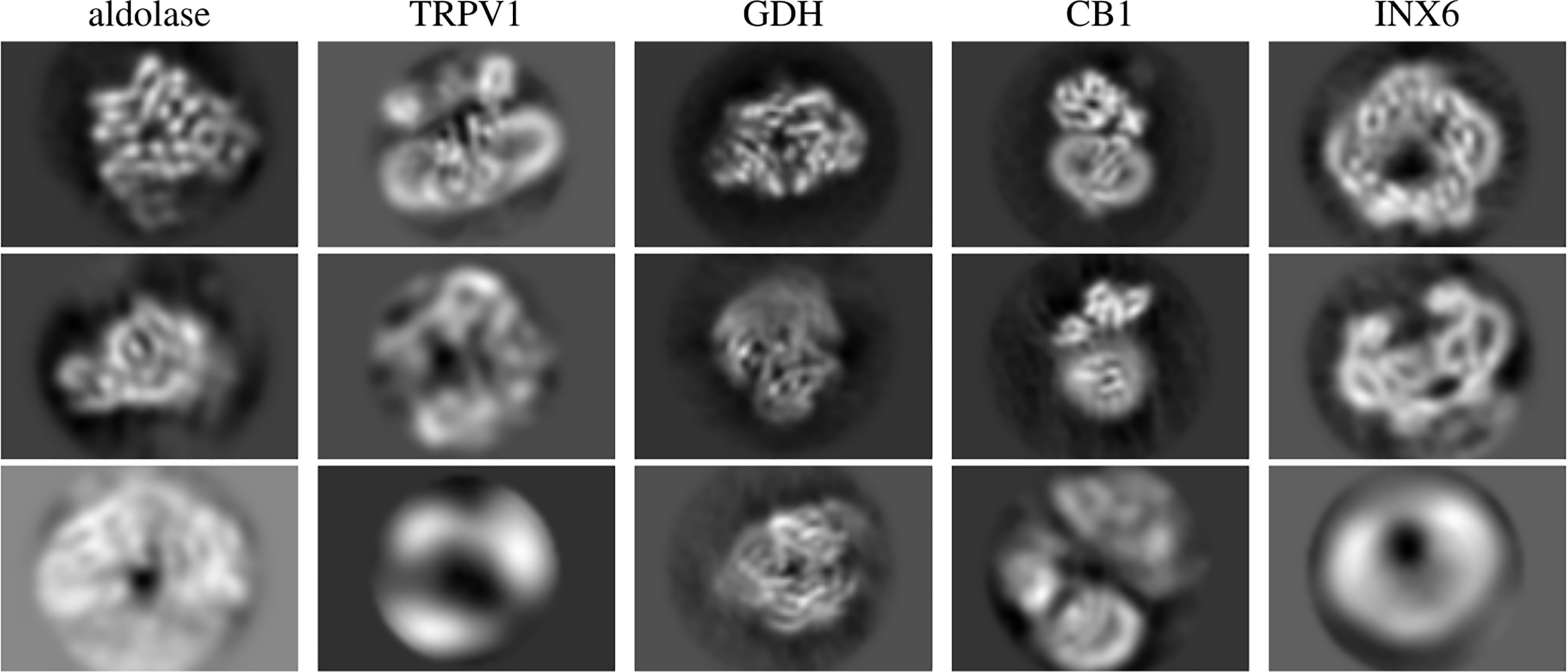
Central slices of initial model reconstruction with three classes using VDAM algorithm for five data sets.

To evaluate the overall performance of the VDAM algorithm for 3D initial model generation, we performed ten repeats of 3D initial model jobs for the particle sets that were selected automatically by the relion_it.py approach of the five data sets shown in Figure 8. Following the relion_it.py approach in RELION-4.0, we used the VDAM algorithm with three classes and selected the most populated class after 200 iterations. For each run, we used the selected model as initial reference for subsequent auto-refinement, and used FSC of the refined structure to the known target structure, after manual alignment in Chimera [49], as a metric to distinguish between successful and unsuccessful runs.

Figure 8 shows examples of central slices of initial model reconstructions that converged to the target structures for the five data sets. For the GDH, CB1 and INX6 data sets, initial model generation was successful for 8, 9 and 10 out of the 10 runs performed, respectively. For the aldolase data sets, 5 out of the 10 VDAM calculations were successful. Convergence within 200 iterations was primarily hindered by heterogeneity, since a large subset of the data set consisted of similar but incomplete particles. Since the automated procedure picks the most populated class, sometimes the target structure is missed as it comes in at second place. The target structure, exhibiting D2 symmetry, makes up about 50% of the data set after the automated 2D class selection. Manual selection might be a necessity to acquire a good final reconstruction for this data set. For the TRPV1 data set, only 4 out of 10 runs were successful. In this case, angular alignment posed the primary issue, possibly due to the relatively large membrane patch.

## Discussion

In this paper, we present new features for single-particle analysis in RELION-4.0: the VDAM algorithm for image refinement, the relion_class_ranker program for automated 2D class selection, and the Schemes framework with an updated relion_it.py approach for unsupervised execution of workflows. In addition, we describe the separately distributed MDCatch program that collects metadata from the microscope to facilitate on-the-fly data processing. The RELION-4.0 release also introduces new approaches to sub-tomogram averaging, and a tighter integration of its pipeline approach with the CCP-EM software suite [50]. These two developments will be described elsewhere.

By iterating through the data set fewer times, the VDAM algorithm provides substantial increases in speed compared with the EM algorithm. For example, we illustrate that 2D classification with the VDAM algorithm is up to six times faster than the EM algorithm, with larger gains in speed observed for larger data sets. Even larger speed-ups may be obtained by reducing the default number of mini-batches from 200 to 100. Although performing fewer iterations may affect the quality of the results for difficult data sets, the additional speed-ups obtained might be valuable for better behaved data sets.

Besides speed improvements, our VDAM implementation also replaces inactive classes, which typically leads to higher numbers of suitable classes that better capture the heterogeneity in the data. Compared with the standard SGD algorithm, the VDAM algorithm also shows improved convergence behavior for 3D initial model generation. Because the VDAM algorithm automatically determines the regularization parameters, 3D initial model calculations with the VDAM algorithm no longer need to be explicitly limited in resolution, as was the case with the SGD algorithm. Thereby, higher resolution initial models may be calculated without user intervention, which leads to more straightforward selection of suitable initial models. The freedom to progress to higher resolutions may also contribute to better convergence of the VDAM algorithm compared with standard SGD. Although not illustrated here, the VDAM algorithm can also be used for 3D classification and 3D auto-refinement. The latter applications may be particularly interesting in the context of injecting more prior knowledge about protein structures into the 3D reconstruction process [51], which will be a direction of future research.

In previous release of RELION, manual selection of suitable 2D class average images represented a hurdle for automated on-the-fly image processing in the typical workflow. The relion_class_ranker program overcomes this hurdle. We found that a combination of a feature vector with a convolutional neural network that acts on the 2D class average images yields excellent results in predicting scores for 2D classes that allow their unsupervised selection. The feature vector contains two types of features. On the one hand, features like the estimated angular and translational alignment accuracy and the class sizes contribute information from the RELION refinement process that is not present in the class average images. On the other hand, hand-crafted features that are calculated from the class average images, such as moments of pixel values in protein and solvent masks, allow biasing the scores on information that is assumed to be important.

The class scores from the relion_class_ranker program are on an absolute scale. Although a default selection threshold of 0.15 allowed automated structure determination for eleven out of 12 test data sets, in practice many users may choose to tune the threshold value for their specific type of data. Tuned thresholds gave particle selections with FPRs and FNRs of less than 0.25 for all data sets tested. Ordering 2D class averages by their predicted class scores may also be useful for manual selection. In previous releases of RELION, 2D class average images were typically displayed sorted on their class size. However, the VDAM algorithm often converges to solutions that also contain relatively large classes with suboptimal particles. Therefore, we have observed that sorting the classes based on their predicted scores is also helpful for manual selection of suitable 2D classes. Executing relion_class_ranker typically takes less than a minute.

The development of Schemes for the automated execution of pre-defined, image processing workflows that represent branched decision trees is a less visible part of the improvements in RELION-4.0. As an example of what is possible, we distribute the prep and proc Schemes for automated structure determination inside the new relion_it.py approach. Although we show the usefulness of this fully automated approach on 12 test data sets, we expect that many users will want to modify parts of this approach to fit their specific needs. The Schemes are aimed at providing the flexibility that will be required by the different types of end-users to automate a wide range of image processing tasks.

In general, as cryo-EM structure determination continues to improve rapidly, we envision that flexibility in the design of image processing workflows will remain essential for many users. The RELION tools described here aim to facilitate this flexibility, as well as speed, and to help the inexperienced user in getting the most of their cryo-EM images, while at the same time providing the advanced user with all the tools necessary to solve the most difficult structures. Moreover, by distributing these tools as free, open-source software, we encourage the cryo-EM community to build on the advances described.

## Data Availability

RELION-4.0 is distributed under a GPLv2 license and can be downloaded for free from https://github.com/3dem/relion/tree/ver4.0. The 2D class average images used for training the neural network in the relion_class_ranker, together with all necessary metadata to replicate the training, can be downloaded from the EMPIAR data base (entry-ID 10812).

## Abbreviations

CTF: contrast transfer function
FNR: false negative rate
FPR: false positive rate
LoG: Laplacian-of-Gaussian
MAP: maximum a posteriori
VMGD: variable-metric gradient descent

## References

[1] Yip K.M., Fischer N., Paknia E., Chari A. and Stark H. (2020) Atomic-resolution protein structure determination by cryo-EM. Nature 587, 157–161 10.1038/s41586-020-2833-4 33087927

[2] Nakane T., Kotecha A., Sente A., McMullan G., Masiulis S. and Brown P.M. et al. (2020) Single-particle cryo-EM at atomic resolution. Nature 587, 152–156 10.1038/s41586-020-2829-0 33087931 PMC7611073

[3] Frank J., Radermacher M., Penczek P., Zhu J., Li Y., Ladjadj M. et al. (1996) SPIDER and WEB: processing and visualization of images in 3D electron microscopy and related fields. J. Struct. Biol. 116, 190–199 10.1006/jsbi.1996.0030 8742743

[4] van Heel M., Harauz G., Orlova E.V., Schmidt R. and Schatz M. (1996) A new generation of the imagic image processing system. J. Struct. Biol. 116, 17–24 10.1006/jsbi.1996.0004 8742718

[5] Crowther R., Henderson R. and Smith J.M. (1996) MRC image processing programs. J. Struct. Biol. 116, 9–16 10.1006/jsbi.1996.0003 8742717

[6] Ludtke S.J., Baldwin P.R. and Chiu W. (1999) EMAN: semiautomated software for high-resolution single-particle reconstructions. J. Struct. Biol. 128, 82–97 10.1006/jsbi.1999.4174 10600563

[7] Hohn M., Tang G., Goodyear G., Baldwin P.R., Huang Z., Penczek P.A. et al. (2007) SPARX, a new environment for cryo-EM image processing. J. Struct. Biol. 157, 47–55 10.1016/j.jsb.2006.07.003 16931051

[8] Heymann J.B. (2001) Bsoft: image and molecular processing in electron microscopy. J. Struct. Biol. 133, 156–169 10.1006/jsbi.2001.4339 11472087

[9] Tang G., Peng L., Baldwin P.R., Mann D.S., Jiang W., Rees I. et al. (2007) EMAN2: an extensible image processing suite for electron microscopy. J. Struct. Biol. 157, 38–46 10.1016/j.jsb.2006.05.009 16859925

[10] Scheres S.H., Núñez-Ramírez R., Sorzano C.O., Carazo J.M. and Marabini R. (2008) Image processing for electron microscopy single-particle analysis using XMIPP. Nat. Protoc. 3, 977–990 10.1038/nprot.2008.62 18536645 PMC2778070

[11] Kühlbrandt W. (2014) The resolution revolution. Science 343, 1443–1444 10.1126/science.1251652 24675944

[12] Moriya T., Saur M., Stabrin M., Merino F., Voicu H., Huang Z. et al. (2017) High-resolution single particle analysis from electron cryo-microscopy images using SPHIRE. J. Vis. Exp. (JoVE) 123, e55448 10.3791/55448 PMC5607996 28570515

[13] Grant T., Rohou A. and Grigorieff N. (2018) *cis*TEM, user-friendly software for single-particle image processing. eLife 7, e35383 10.7554/eLife.35383 29513216 PMC5854467

[14] Punjani A., Rubinstein J.L., Fleet D.J. and Brubaker M.A. (2017) cryoSPARC: algorithms for rapid unsupervised cryo-EM structure determination. Nat. Methods 14, 290–296 10.1038/nmeth.4169 28165473

[15] Lander G.C., Stagg S.M., Voss N.R., Cheng A., Fellmann D. and Pulokas J. et al. (2009) Appion: an integrated, database-driven pipeline to facilitate EM image processing. J. Struct. Biol. 166, 95–102 10.1016/j.jsb.2009.01.002 19263523 PMC2775544

[16] De la Rosa-Trevín J., Quintana A., Del Cano L., Zaldívar A., Foche I. and Gutiérrez J. et al. (2016) Scipion: a software framework toward integration, reproducibility and validation in 3D electron microscopy. J. Struct. Biol. 195, 93–99 10.1016/j.jsb.2016.04.010 27108186

[17] Scheres S.H. (2012b) RELION: implementation of a Bayesian approach to cryo-EM structure determination. J. Struct. Biol. 180, 519–530 10.1016/j.jsb.2012.09.006 23000701 PMC3690530

[18] Scheres S.H. (2012) A Bayesian view on cryo-EM structure determination. J. Mol. Biol. 415, 406–418 10.1016/j.jmb.2011.11.010 22100448 PMC3314964

[19] Patwardhan A. (2017) Trends in the electron microscopy data bank (EMDB). Acta Crystallogr. D: Struct. Biol. 73, 503–508 10.1107/S2059798317004181 28580912 PMC5458492

[20] Biyani N., Righetto R.D., McLeod R., Caujolle-Bert D., Castano-Diez D., Goldie K.N. et al. (2017) Focus: the interface between data collection and data processing in cryo-EM. J. Struct. Biol. 198, 124–133 10.1016/j.jsb.2017.03.007 28344036

[21] Gómez-Blanco J., Marabini R., Del Cano L., Jiménez A., Martínez M. and Melero R. et al. (2018) Using scipion for stream image processing at cryo-EM facilities. J. Struct. Biol. 204, 457–463 10.1016/j.jsb.2018.10.001 30296492 PMC6303188

[22] Tegunov D. and Cramer P. (2019) Real-time cryo-electron microscopy data preprocessing with Warp. Nat. Methods 16, 1146–1152 10.1038/s41592-019-0580-y 31591575 PMC6858868

[23] Stabrin M., Schoenfeld F., Wagner T., Pospich S., Gatsogiannis C. and Raunser S. (2020) TranSPHIRE: automated and feedback-optimized on-the-fly processing for cryo-EM. Nat. Commun. 11, 1–14 10.1038/s41467-020-19513-2 33177513 PMC7658977

[24] Xie R., Chen Y.-X., Cai J.-M., Yang Y. and Shen H.-B. (2020) Spread: a fully automated toolkit for single-particle cryogenic electron microscopy data 3D reconstruction with image-network-aided orientation assignment. J. Chem. Inf. Model. 60, 2614–2625 10.1021/acs.jcim.9b01099 31990536

[25] Schenk A.D., Cavadini S., Thoma N.H. and Genoud C. (2020) Live analysis and reconstruction of single-particle cryo-electron microscopy data with cryoflare. J. Chem. Inf. Model. 60, 2561–2569 10.1021/acs.jcim.9b01102 32233514

[26] Xu L. and Jordan M.I. (1996) On convergence properties of the EM algorithm for gaussian mixtures. Neural Comput. 8, 129–151 10.1162/neco.1996.8.1.129 11112259

[27] Kingma D.P. and Ba J. (2014) Adam: a method for stochastic optimization. Preprint arXiv:1412.6980.

[28] Duchi J., Hazan E. and Singer Y. (2011) Adaptive subgradient methods for online learning and stochastic optimization. J. Mach. Learn. Res. 12, 2121–2159 https://www.jmlr.org/papers/volume12/duchi11a/duchi11a.pdf

[29] Zeiler M.D. (2012) ADADELTA: an adaptive learning rate method. Preprint arXiv:1212.5701.

[30] Loshchilov I. and Hutter F. (2017) Decoupled weight decay regularization. Preprint arXiv:1711.05101.

[31] Martinetz T. and Schulten K. (1991) A ‘neural-gas’ network learns topologies. In *Artificial Neural Networks* (Kohonen, T., Makisara, K., Simula, O. and Kangas, J., eds), Elsevier Science Publisher, B.V. North-Holland

[32] Paszke A., Gross S., Massa F., Lerer A., Bradbury J. and Chanan G. (2019) PyTorch: an imperative style, high-performance deep learning library. In *Advances in Neural Information Processing Systems 32* (Wallach, H., Larochelle, H., Beygelzimer, A., d’Alché-Buc, F., Fox, E. and Garnett, R., eds), pp. 8024–8035, Curran Associates, Inc, Red Hook, NY, US.

[33] Fernandez-Leiro R. and Scheres S.H. (2017) A pipeline approach to single-particle processing in RELION. Acta Crystallogr. D: Struct. Biol. 73, 496–502 10.1107/S2059798316019276 28580911 PMC5458491

[34] Bepler T., Kelley K., Noble A.J. and Berger B. (2020) Topaz-Denoise: general deep denoising models for cryoEM and cryoET. Nat. Commun. 11, 1–12 10.1038/s41467-020-18952-1 33060581 PMC7567117

[35] Mastronarde D.N. (2005) Automated electron microscope tomography using robust prediction of specimen movements. J. Struct. Biol. 152, 36–51 10.1016/j.jsb.2005.07.007 16182563

[36] Wagner T., Merino F., Stabrin M., Moriya T., Antoni C., Apelbaum A. et al. (2019) SPHIRE-crYOLO is a fast and accurate fully automated particle picker for cryo-EM. Commun. Biol. 2, 218 10.1038/s42003-019-0437-z 31240256 PMC6584505

[37] Bepler T., Morin A., Rapp M., Brasch J., Shapiro L., Noble A.J. et al. (2019) Positive-unlabeled convolutional neural networks for particle picking in cryo-electron micrographs. Nat. Methods 16, 1153–1160 10.1038/s41592-019-0575-8 31591578 PMC6858545

[38] Iudin A., Korir P.K., Salavert-Torres J., Kleywegt G.J. and Patwardhan A. (2016) EMPIAR: a public archive for raw electron microscopy image data. Nat. Methods 13, 387–388 10.1038/nmeth.3806 27067018

[39] Wong W., Bai X.-c., Brown A., Fernandez I.S., Hanssen E., Condron M. et al. (2014) Cryo-EM structure of the *Plasmodium falciparum* 80S ribosome bound to the anti-protozoan drug emetine. eLife 3, e03080 10.7554/eLife.03080 PMC4086275 24913268

[40] Gao Y., Cao E., Julius D. and Cheng Y. (2016) TRPV1 structures in nanodiscs reveal mechanisms of ligand and lipid action. Nature 534, 347–351 10.1038/nature17964 27281200 PMC4911334

[41] von Loeffelholz O., Papai G., Danev R., Myasnikov A.G., Natchiar S.K., Hazemann I. et al. (2018) Volta phase plate data collection facilitates image processing and cryo-EM structure determination. J. Struct. Biol. 202, 191–199 10.1016/j.jsb.2018.01.003 29337113

[42] Herzik M.A., Wu M. and Lander G.C. (2017) Achieving better-than-3-Å resolution by single-particle cryo-EM at 200 keV. Nat. Methods 14, 1075–1078 10.1038/nmeth.4461 28991891 PMC5679434

[43] Bai X.-C., Yan C., Yang G., Lu P., Ma D., Sun L. et al. (2015) An atomic structure of human *γ*-secretase. Nature 525, 212–217 10.1038/nature14892 26280335 PMC4568306

[44] Thompson R.F., Iadanza M.G., Hesketh E.L., Rawson S. and Ranson N.A. (2019) Collection, pre-processing and on-the-fly analysis of data for high-resolution, single-particle cryo-electron microscopy. Nat. Protoc. 14, 100–118 10.1038/s41596-018-0084-8 30487656 PMC7618007

[45] Kumar K.K., Shalev-Benami M., Robertson M.J., Hu H., Banister S.D. and Hollingsworth S.A. et al. (2019) Structure of a signaling cannabinoid receptor 1-g protein complex. Cell 176, 448–458 10.1016/j.cell.2018.11.040 30639101 PMC6461403

[46] Burendei B., Shinozaki R., Watanabe M., Terada T., Tani K., Fujiyoshi Y. et al. (2020) Cryo-EM structures of undocked innexin-6 hemichannels in phospholipids. Sci. Adv. 6, eaax3157 10.1126/sciadv.aax3157 32095518 PMC7015682

[47] Zivanov J., Nakane T., Forsberg B.O., Kimanius D., Hagen W.J., Lindahl E. et al. (2018) New tools for automated high-resolution cryo-EM structure determination in RELION-3. eLife 7, e42166 10.7554/eLife.42166 30412051 PMC6250425

[48] Rohou A. and Grigorieff N. (2015) ctffind4: fast and accurate defocus estimation from electron micrographs. J. Struct. Biol. 192, 216–221 10.1016/j.jsb.2015.08.008 26278980 PMC6760662

[49] Pettersen E.F., Goddard T.D., Huang C.C., Couch G.S., Greenblatt D.M., Meng E.C. et al. (2004) UCSF Chimera: a visualization system for exploratory research and analysis. J. Comput. Chem. 25, 1605–1612 10.1002/(ISSN)1096-987X 15264254

[50] Burnley T., Palmer C.M. and Winn M. (2017) Recent developments in the CCP-EM software suite. Acta Crystallogr. D: Struct. Biol. 73, 469–477 10.1107/S2059798317007859 28580908 PMC5458488

[51] Kimanius D., Zickert G., Nakane T., Adler J., Lunz S. and Schönlieb C.-B. et al. (2021) Exploiting prior knowledge about biological macromolecules in cryo-EM structure determination. IUCrJ 8, 60–75 10.1107/S2052252520014384 PMC7793004 33520243

